# Effects of multiparity and prolonged breast-feeding on maternal bone mineral density: a community-based cross-sectional study

**DOI:** 10.1186/1472-6874-9-19

**Published:** 2009-07-01

**Authors:** Janaka Lenora, Sarath Lekamwasam, Magnus K Karlsson

**Affiliations:** 1 Department of Physiology, Faculty of Medicine, University of Ruhuna, Galle, Sri Lanka; 2 Clinical and Molecular Osteoporosis Research Unit, Department of Orthopedics, Department of Clinical Sciences, Lund University, Malmö University Hospital, Malmö, Sweden; 3 Centre for Metabolic Bone Diseases, Department of Medicine, Faculty of Medicine, University of Ruhuna, Galle, Sri Lanka

## Abstract

**Background:**

Studies conducted in Western countries have shown that bone loss associated with pregnancy and breast-feeding is recovered after weaning. However, it is not clear whether recovery takes place after repeated pregnancies followed by prolonged periods of breast-feeding; especially in developing countries where nutritional intake is comparatively low.

This study was designed to examine the effects of multiparity and prolonged breast-feeding on maternal bone mineral density (BMD) in a community-based sample of 210 Sri Lankan women, aged between 46 and 98 years.

**Methods:**

BMD of the lumbar spine (L_2_–L_4_) and femoral neck were measured by dual-energy X-ray absorptiometry. Reproductive history was recorded by using a questionnaire. Women were, first, divided into groups according to parity (nulliparous, 1–2, 3–4, and 5 or more children), and BMDs in different groups were compared, initially unadjusted and then adjusted for age. Same subjects were subdivided, again, according to the total duration of breast-feeding (0, 1–48, 49–96, and 97 months or more) and similar analysis was carried out.

**Results:**

Women who had 5 or more children and women who had breast-fed for 97 months or more were older than the other women (p < 0.01) but no differences in height, weight or BMI were observed among the groups. Age adjusted BMD at lumbar spine and femoral neck BMDs of women grouped according to parity were not significantly different. Neither was there any difference between lumbar spine or femoral neck BMD in groups based on duration of breast-feeding.

**Conclusion:**

From this population-based study conducted in a developing country, we infer that history of multiparity or prolonged breast-feeding has no detrimental effects on maternal BMD in post-menopausal age.

## Background

Both pregnancy and breast-feeding are associated with changes in maternal calcium homeostasis, resulting in decreased bone mineral density (BMD) [1-3]. During pregnancy, approximately 25–30 g calcium, corresponding to 2–3% of the total body calcium content of the mother is transferred to the fetus. The greatest transfer takes place during the second and third trimesters when fetal bone development peaks [1]. If a pregnancy is followed by a period of breast-feeding, mother loses a further 300–400 mg calcium daily in the breast milk [2,3]. Although endocrine changes induce compensatory mechanisms, such as increased intestinal calcium absorption [4], and renal conservation of calcium [5] to counteract the calcium loss, studies have shown that postpartum women have 2–9% lower BMD than matched controls [6,7]. A six-month breast-feeding period is associated with a further 1–6% loss in maternal BMD [8-12] and pregnancy-related osteoporosis has been reported [13].

Longitudinal studies have shown that bone loss associated with pregnancy and breast-feeding is usually recovered after weaning [11,14,15], however it is not clear whether this bone loss is completely recovered in women who have borne many children or in women with a longer total duration of breast-feeding. Current evidence has arisen from studies conducted in developed counties, where frequent pregnancies and prolonged breast-feeding are relatively uncommon, and where the nutritional intake is usually adequate. Fewer studies have been carried out in developing countries where nutritional intake, including calcium and vitamin D, is poor and where multiple pregnancies are common. This raises the possibility that multiple pregnancies and prolonged breast-feeding, could be a risk factor for low BMD, in developing countries. This study was therefore designed to evaluate the effects of repeated pregnancy and prolonged breast-feeding on BMD in a cohort of postmenopausal women from a rural community setting in Sri Lanka.

## Methods

### Study sample

This study was performed as a part of a population-based osteoporosis study in the Community Study Area of the Faculty of Medicine, University of Ruhuna, Galle, Sri Lanka. Four hundred and fifty women aged 30 years or more, selected randomly using the latest electoral register, (2002) were considered to be eligible for the study. All the women were invited to the Centre for Metabolic Bone Diseases, Galle and three hundred and forty three women attended (response rate = 76%). One hundred and seven women did not attend even after several reminders due to unwillingness to participate, leaving the area, bad health or family commitments. Women with history of diseases such as inflammatory arthritis, inflammatory bowel disease, chronic asthma, hyperthyroidism, Cushing's syndrome, type I diabetes and hyperparathyroidism were excluded from the study. Women who had taken medications such as bisphosphanates, hormone replacement therapy, oral corticosteroids, thyroxin, vitamin D preparations, pharmacological doses of calcium, and thiazide diuretics were also excluded. After these exclusions, 328 women remained, and of them, all postmenopausal women (n = 210) were eligible for inclusion in this study. These women were interviewed using a detailed investigator-administered questionnaire, to gather demographic data, medical history, and reproductive history, including number of pregnancies, number of live births, duration of breast-feeding for each child and age of menopause.

### Bone mineral density (BMD)

BMD was measured at the spine from the second to fourth lumbar vertebrae (L_2_–L_4_), antero-posterior projection, and in the non-dominant proximal femur (femoral neck) using dual-energy X-ray absorbtiometry (DXA) (Norland Eclipse XR, Norland Corp., Ford Atkinson, WI, USA). The coefficient of variation (CV%) of the DXA equipment as assessed by duplicate measurements of 30 women after repositioning between two scans, was 1.0% for L_2_–L_4 _and 1.5% for the femoral neck.

### Anthropometry

Standing height was measured without footwear and recorded to the nearest mm (Stadiometer^® ^Yamuchi), weight was measured to the nearest 0.1 kg using a beam balance (Bauman^®^, Germany) while wearing light cloths and no shoes. From these, body mass index (BMI) was then calculated (body weight/height^2^).

The study was approved by the Ethical Review Committee of the Faculty of Medicine, University of Ruhuna, Galle, Sri Lanka, and was performed according to the declaration of Helsinki 2000. Informed, consent was obtained from each of the participants prior to the study

### Statistical analysis

Statistica for Windows (version 7.1, Stat Soft Inc.) was used for the statistical analysis. Number of children and the total period of breast feeding showed a skewed distribution. The women were divided, initially, into groups according to parity (nulliparous, 1–2 children, 3–4 children, 5 or more children), and then according to the total duration of breast-feeding (never breast-fed, 1–48 months, 49–96 months, more than 97 months). When a child was reported to have been breast-fed for more than 24 months, a maximum of 24 months was recorded as it was assumed that intermittent breast-feeding beyond 24 months had no substantial effect on maternal bone metabolism, because almost all children, by 2 years of age, were weaned and receiving meals at least three times a day. In the case of women with more than one child, the durations of breast-feeding for each child were summed up to calculate the total duration of breast feeding.

Analysis of variance (ANOVA) was used to compare the different groups. Analysis of covariance (ANCOVA) was used to adjust for differences in age, age of menopause, duration since menopause, BMI and duration of breast-feeding or number of childbirths. Data are presented as means and 95% confidence intervals (95% CI) unless stated otherwise.

## Results

### Basic characteristics

The participants ranged from 45.8–97.7 years of age, with a mean (standard deviation) of 64.6 (8.7) years. Mean (SD) age at menopause and duration since menopause of participants were 47.8 (4.1) years and 16.6 (9.5) years respectively. Of the 210 women included in the study, 35 had never been pregnant. The number of childbirths ranged from 0 to 10 (median = 4, inter-quartile range = 3–5). Only one woman who had borne a child had failed to breast-feed. The total duration of breast-feeding ranged from 0 to 216 months (median = 72, inter-quartile range = 42–102 months). Of the participants, 137 women (65.2%) had more than 3 children and 106 women (50.5%) had breast-fed for a total of more than 49 months. 170 women had never been employed while 27 were unskilled manual workers, and only 13 had jobs with regular income. None of the subjects had ever smoked or taken alcohol during their life time. Only 4 (1.9%) women had used hormonal contraceptive methods.

### Effect of parity

Women who had borne 5 children or more were older than the other women (p < 0.001) and had a longer duration since menopause (p < 0.01) (Table 1). There were no differences in height, weight, or BMI between the groups of women with different parity (Table 1). Women who had borne 5 children or more had a longer total duration of breast-feeding than women with 1–2 or 3–4 children, and women with 3–4 children had a longer total duration of breast-feeding than women with 1–2 children (both p < 0.001) (Table 1).

**Table 1:** Basic characteristics and bone mineral density (BMD) in the cohort of postmenopausal women, grouped according to number of children borne.

	**All women** **(n = 210)**	**Number of children borne**				
		
		**None (n = 35)**	**1–2 (n = 38)**	**3–4 (n = 70)**	**≥ 5 (n = 67)**	**p value**
Age (years)	64.6 (63.4–65.8)	62.4 (59.9–64.8)	64.4 (61.4–67.4)	62.5 (60.5–64.6)	68.1 (66.1–70.1)	**< 0.001**

Age of menopause	47.8 (47.2–48.4)	47.1 (45.6–48.6)	48.0 (46.7–49.3)	48.0 (47.0–49.0)	47.7 (46.7–48.8)	**0.79**

Duration since menopause	16.6 (15.3–17.9)	14.4 (11.0–17.7)	16.4 (13.4–19.3)	14.4 (12.2–16.6)	20.1 (17.8–22.4)	**< 0.01**

Height (m)	1.47 (1.46–1.48)	1.48 (1.46–1.50)	1.47 (1.45–1.49)	1.47 (1.46–1.49)	1.46 (1.45–1.47)	0.47

Weight (kg)	45.6 (44.3–46.9)	43.4 (39.8–46.9)	45.8 (43.1–48.5)	46.7 (44.3–49.1)	45.6 (43.4–47.8)	0.41

Body mass index (BMI; kg/m^2^)	21.2 (20.6–21.7)	19.8 (18.3–21.4)	21.2 (20.1–22.3)	21.6 (20.5–22.6)	21.4 (20.4–22.4)	0.216

Total duration of breast-feeding (months)	61.3 (54.5–68.2)	-	28.6 (23.3–33.8)	59.2 (53.2–65.1)	114.6 (104.0–124.5)	**< 0.001**

Children (n)	3.5 (3.2–3.9)	0	1.6 (1.4–1.8)	3.5 (3.4–3.7)	6.4 (6.2–6.7)	-

L_2_–L_4 _vertebrae BMD (g/cm^2^)	0.688 (0.668–0.707)	0.673 (0.630–0.716)	0.693 (0.650–0.737)	0.698 (0.667–0.729)	0.681 (0.649–0.714)	0.77

Femoral neck BMD (g/cm^2^)	0.628 (0.612–0.644)	0.613 (0.580–0.646)	0.648 (0.616–0.680)	0.637 (0.614–0.661)	0.614 (0.589–0.639)	0.24

Data are given as mean (95% confidence intervals) of each group with a p-value for the general trend.

BMD data presented are adjusted for age.

No differences were found in age-adjusted BMD when comparing groups of women with different parity (Table 1). These results remained unchanged when adjustments were made for age, age of menopause, duration since menopause, BMI, and total duration of breast-feeding (data not shown). Although there was a difference in duration since menopause among the groups, it's effect was masked by the effect of age. Data were not adjusted for smoking habits and alcohol consumption as this was not applicable to our subjects.

### Effect of breast-feeding

Women who had breast-fed for a total period of >97 months were older than women with shorter total periods of breast-feeding (p < 0.001) and had a longer duration since menopause (p < 0.001) (Table 2). There were no differences in height, weight, or BMI between groups with different total breast-feeding duration (Table 2). An association was found between number of children and total duration of breast-feeding (all group comparisons p < 0.001) (Table 2).

**Table 2:** Basic characteristics and bone mineral density (BMD) in women, grouped according to total duration of breast-feeding.

	**Duration of breast-feeding (months)**				
	**Never (n = 36)**	**1–48 months (n = 68)**	**49–96 months (n = 61)**	**≥ 97 months (n = 45)**	**p value**

Age (years)	62.6 (59.9–65.3)	62.8 (60.8–64.8)	64.3 (62.2–66.4)	69.4 (67.0–71.8)	**< 0.001**

Height (m)	1.48 (1.46–1.50)	1.47 (1.46–1.49)	1.46 (1.45–1.48)	1.46 (1.44–1.48)	0.48

Age of menopause (years)	47.2 (45.8–48.7)	47.6 (46.6–48.6)	48.3 (47.2–49.3)	47.8 (46.6–49.1)	**0.69**

Duration since menopause (years)	14.6 (11.3–17.8)	15.1 (12.9–17.3)	15.5 (13.2–17.9)	21.7 (19.0–24.5)	**< 0.001**

Weight (kg)	43.4 (40.3–46.5)	46.1 (43.9–48.4)	46.8 (44.5–49.2)	45.0 (42.3–47.8)	0.33

Body mass index (BMI; kg/m^2^)	19.8 (18.3–21.2)	21.3 (20.3–22.3)	21.8 (20.8–22.8)	21.1 (19.1–22.4)	0.16

Total period of breast-feeding (months)	-	31.5 (27.1–35.9)	75.6 (71–80.2)	136.2 (130.8–141.5)	-

Children (n)	0	2.5 (2.2–2.8)	4.3 (4.0–4.6)	6.8 (6.4–7.2)	**< 0.001**

L_2_–L_4 _vertebrae BMD (g/cm^2^)	0.670 (0.628–0.711)	0.694 (0.661–0.726)	0.709 (0.676–0.742)	0.666 (0.627–0.705)	0.31

Femoral neck BMD (g/cm^2^)	0.613 (0.580–0.646)	0.643 (0.620–0.668)	0.637 (0.611–0.662)	0.603 (0.573–0.634)	0.15

Data are given as mean (95% confidence interval) of each group with a p-value for the general trend.

BMD data presented are adjusted for age.

No differences were found in age-adjusted BMD when groups of women with different total durations of breast-feeding were compared (Table 2). These results remained unchanged when adjustments were made for age, age of menopause, duration since menopause, BMI, and number of children (data not shown).

## Discussion

The results of this community-based cross-sectional study, carried out on rural Sri Lankan postmenopausal women, indicate that repeated childbearing and breast-feeding for long periods is not associated with low femoral neck or lumbar spine BMD. It is difficult to differentiate between the effects of childbearing and breast-feeding due to the strong correlation between the two. In a clinical situation, however, they can be regarded as a combined risk factor. As no differences in the age-adjusted lumbar spine and femoral neck BMD were found in the subgroups, it can be inferred that the combined effect of number of pregnancies and duration of breast-feeding should not be considered a risk factor for low BMD in these postmenopausal women.

During pregnancy and breast-feeding, substantial changes take place in bone metabolism, leading to the hypothesis that pregnancy and breast-feeding may be risk factors for low BMD. During pregnancy, maternal fractional calcium absorption in the gut is increased by 50%, as a result of increased levels of 1,25 dihydroxy-vitamin D [4,5] and estrogen levels in the maternal circulation are high. These both offer protection against bone calcium loss. The maternal parathyroid hormone (PTH) level which is also high during pregnancy, causes mobilization of calcium from the skeleton [16]. Furthermore, in spite of increased renal tubular calcium reabsorption, mediated via 1,25 dihydroxy-vitamin D, the urinary excretion of calcium is still high due to the high glomerular filtration rate [2,17]. The mechanism controlling demineralization of the maternal skeleton during breast-feeding is not well understood [18,19], but studies suggest that bone loss may be partly mediated by parathormone-related peptide (PTHrP) and low estrogen levels during the period of lactational amenorrhea [2]. Despite the body's attempts to maintain calcium homeostasis, maternal BMD decreases on average by 5% during pregnancy and breast-feeding [6]. However, nutritional intake, which is often higher in pregnant and lactating women than other women [20], as well as changes in the level of physical activity, may also play a role in the regulation of maternal BMD.

The question then arises as to whether the changes in bone metabolism in pregnant and lactating woman lead to changes in maternal BMD in the longer perspective. Only a few studies have reported the effects of parity on BMD using a population of postmenopausal women with high parity. Studies conducted in American [21,22] and Japanese [23] populations suggest that multiparity has no long-term beneficial or detrimental effects on maternal BMD. This view is, however, opposed by others and two studies in Turkish postmenopausal women revealed low spinal BMD in women who had borne more than 5 children [24] and that high parity is a risk factor for low spinal BMD and osteoporosis [25]. Allali *et al *found low spinal and total hip BMD in Moroccan postmenopausal women with 6 or more children [26]. The long term clinical relevance of low BMD associated with multiparity is also unclear since there are reports that women with multiple pregnancies have a lower fracture risk than nulliparous women [27,28], and that the risk of fracture is reduced with each child delivered [29]. Extended breast-feeding has also been reported to be associated with reduced risk of fracture in Chinese women [30].

Few studies have evaluated the long-term effect of breast feeding on the maternal skeleton [22,31-33]. A study conducted on Turkish women reported that a longer period of breast feeding was associated with lower BMD in the lumbar spine and femoral neck, and it was concluded that prolonged breast-feeding is a risk factor for osteoporosis in postmenopausal women [32]. However, others have found that the total duration of breast-feeding is not associated with reduced age-adjusted BMD in groups of American [22,33] and Japanese women [23]. Chowdhury *et al *also reported, in a sample of Bangladeshi women aged 20–81 years, that the negative correlation found between the duration of breast-feeding and BMD in the ultra-distal fore-arm, was dependent on other co-variants such as age, BMI and lactational amenorrhea [31].

Compared to previous studies [32,33], one of the strengths of the present study is that it included women with a much longer total duration of breast feeding and as such has the advantage of evaluating postmenopausal women who have never been pregnant or involved in breast-feeding [31]. Most of the previous studies have been carried out in Western countries, or countries where maternal nutrition is usually adequate, and long-term breast-feeding is uncommon. In contrast, the women in this study were from a lower socio-economic background (which often means marginal nutrition), although we have no specific nutritional data with which to define the nutritional status.

Weaknesses of the study include the requirement for the subjects to recall past events and recall bias may have occurred during data collection. The wide range (from 46 to 98 years) of age is also a limiting factor. Although we adjusted for age, other age-related factors such as physical activity may have influenced the results. In this type of community survey however, it is difficult to control for such confounding factors. Another potential limitation of the study is that in the event that a child was reported to be breast fed in excess of 24 months, the effective period was recorded as 24 months assuming that intermittent breast-feeding beyond 24 months had no substantial effect on maternal BMD. As mentioned above, it is difficult to identify, separately, the individual effects of childbearing and breast-feeding, due to the strong relationship between the two. However, adjusting first for age and then age together with BMI and number of pregnancies, or age, BMI and the total duration of breast-feeding, showed that neither the number of pregnancies nor the duration of breast-feeding could be regarded as a risk factor for low postmenopausal BMD.

## Conclusion

In conclusion, this study indicates that there is no long-term detrimental effect of multiple pregnancies and long total duration of breast-feeding on maternal BMD in women within a socio-economic group whose nutritional intake is often marginal. These data are in general agreement with the common view that mothers should breast feed their children as long as they can, due to the well-known positive effects of breast-feeding.

## Competing interests

The authors declare that they have no competing interests.

## Authors' contributions

JL involved in study design, data collection, statistical analysis of data, interpretation of results and manuscript writing. SL involved in study design, data collection, statistical analysis of data, interpretation of results, and supported in manuscript writing. KMK supported in statistical analysis, interpretation of results, and supported in manuscript writing. All authors have approved the final manuscript.

## Authors' information

JL can also be contacted by email at lenora@med.ruh.ac.lk (after November 2009).

## Pre-publication history

The pre-publication history for this paper can be accessed here:



## References

[B1] Pitkin RM (1985). Calcium metabolism in pregnancy and the perinatal period: a review. Am J Obstet Gynecol.

[B2] Kovacs CS (2001). Calcium and bone metabolism in pregnancy and lactation. J Clin Endocrinol Metab.

[B3] Laskey MA, Prentice A, Shaw J, Zachou T, Ceesay SM, Vasquez-Velasquez L, Fraser DR (1990). Breast-milk calcium concentrations during prolonged lactation in British and rural Gambian mothers. Acta Paediatr Scand.

[B4] O'Brien KO, Nathanson MS, Mancini J, Witter FR (2003). Calcium absorption is significantly higher in adolescents during pregnancy than in the early postpartum period. Am J Clin Nutr.

[B5] Ritchie LD, Fung EB, Halloran BP, Turnlund JR, Van Loan MD, Cann CE, King JC (1998). A longitudinal study of calcium homeostasis during human pregnancy and lactation and after resumption of menses. Am J Clin Nutr.

[B6] Karlsson MK, Ahlborg HG, Karlsson C (2005). Female reproductive history and the skeleton-a review. Bjog.

[B7] Oliveri B, Parisi MS, Zeni S, Mautalen C (2004). Mineral and bone mass changes during pregnancy and lactation. Nutrition.

[B8] Laskey MA, Prentice A, Hanratty LA, Jarjou LM, Dibba B, Beavan SR, Cole TJ (1998). Bone changes after 3 mo of lactation: influence of calcium intake, breast-milk output, and vitamin D-receptor genotype. Am J Clin Nutr.

[B9] Karlsson C, Obrant KJ, Karlsson M (2001). Pregnancy and lactation confer reversible bone loss in humans. Osteoporos Int.

[B10] Krebs NF, Reidinger CJ, Robertson AD, Brenner M (1997). Bone mineral density changes during lactation: maternal, dietary, and biochemical correlates. Am J Clin Nutr.

[B11] Kalkwarf HJ, Specker BL (1995). Bone mineral loss during lactation and recovery after weaning. Obstet Gynecol.

[B12] Wardlaw GM, Pike AM (1986). The effect of lactation on peak adult shaft and ultra-distal forearm bone mass in women. Am J Clin Nutr.

[B13] O'Sullivan SM, Grey AB, Singh R, Reid IR (2006). Bisphosphonates in pregnancy and lactation-associated osteoporosis. Osteoporos Int.

[B14] Bezerra FF, Mendonca LM, Lobato EC, O'Brien KO, Donangelo CM (2004). Bone mass is recovered from lactation to postweaning in adolescent mothers with low calcium intakes. Am J Clin Nutr.

[B15] Polatti F, Capuzzo E, Viazzo F, Colleoni R, Klersy C (1999). Bone mineral changes during and after lactation. Obstet Gynecol.

[B16] O'Brien KO, Donangelo CM, Zapata CL, Abrams SA, Spencer EM, King JC (2006). Bone calcium turnover during pregnancy and lactation in women with low calcium diets is associated with calcium intake and circulating insulin-like growth factor 1 concentrations. Am J Clin Nutr.

[B17] Cross NA, Hillman LS, Allen SH, Krause GF, Vieira NE (1995). Calcium homeostasis and bone metabolism during pregnancy, lactation, and postweaning: a longitudinal study. Am J Clin Nutr.

[B18] Kovacs CS, Kronenberg HM (1997). Maternal-fetal calcium and bone metabolism during pregnancy, puerperium, and lactation. Endocr Rev.

[B19] Woodrow JP, Sharpe CJ, Fudge NJ, Hoff AO, Gagel RF, Kovacs CS (2006). Calcitonin plays a critical role in regulating skeletal mineral metabolism during lactation. Endocrinology.

[B20] Takimoto H, Yoshiike N, Katagiri A, Ishida H, Abe S (2003). Nutritional status of pregnant and lactating women in Japan: a comparison with non-pregnant/non-lactating controls in the National Nutrition Survey. J Obstet Gynaecol Res.

[B21] Streeten EA, Ryan KA, McBride DJ, Pollin TI, Shuldiner AR, Mitchell BD (2005). The relationship between parity and bone mineral density in women characterized by a homogeneous lifestyle and high parity. J Clin Endocrinol Metab.

[B22] Kritz-Silverstein D, Barrett-Connor E, Hollenbach KA (1992). Pregnancy and lactation as determinants of bone mineral density in postmenopausal women. Am J Epidemiol.

[B23] Kojima N, Douchi T, Kosha S, Nagata Y (2002). Cross-sectional study of the effects of parturition and lactation on bone mineral density later in life. Maturitas.

[B24] Gur A, Nas K, Cevik R, Sarac AJ, Ataoglu S, Karakoc M (2003). Influence of number of pregnancies on bone mineral density in postmenopausal women of different age groups. J Bone Miner Metab.

[B25] Demir B, Haberal A, Geyik P, Baskan B, Ozturkoglu E, Karacay O, Deveci S (2008). Identification of the risk factors for osteoporosis among postmenopausal women. Maturitas.

[B26] Allali F, Maaroufi H, Aichaoui SE, Khazani H, Saoud B, Benyahya B, Abouqal R, Hajjaj-Hassouni N (2007). Influence of parity on bone mineral density and peripheral fracture risk in Moroccan postmenopausal women. Maturitas.

[B27] Cure-Cure C, Cure-Ramirez P, Teran E, Lopez-Jaramillo P (2002). Bone-mass peak in multiparity and reduced risk of bone-fractures in menopause. Int J Gynaecol Obstet.

[B28] Hillier TA, Rizzo JH, Pedula KL, Stone KL, Cauley JA, Bauer DC, Cummings SR (2003). Nulliparity and fracture risk in older women: the study of osteoporotic fractures. J Bone Miner Res.

[B29] Michaelsson K, Baron JA, Farahmand BY, Ljunghall S (2001). Influence of parity and lactation on hip fracture risk. Am J Epidemiol.

[B30] Huo D, Lauderdale DS, Li L (2003). Influence of reproductive factors on hip fracture risk in Chinese women. Osteoporos Int.

[B31] Chowdhury S, Sarkar NR, Roy SK (2002). Impact of lactational performance on bone mineral density in marginally-nourished Bangladeshi women. J Health Popul Nutr.

[B32] Dursun N, Akin S, Dursun E, Sade I, Korkusuz F (2006). Influence of duration of total breast-feeding on bone mineral density in a Turkish population: does the priority of risk factors differ from society to society?. Osteoporos Int.

[B33] Melton LJ, Bryant SC, Wahner HW, O'Fallon WM, Malkasian GD, Judd HL, Riggs BL (1993). Influence of breastfeeding and other reproductive factors on bone mass later in life. Osteoporos Int.

